# Natural grasslands converted to afforested lands and abandoned lands decreasing soil carbon stability and storage, respectively, in the China’s Loess Plateau

**DOI:** 10.1371/journal.pone.0335924

**Published:** 2025-11-18

**Authors:** Yao Zhong, Yumei Li, Xuemao Zhao

**Affiliations:** College of Geography and Environmental Science, Northwest Normal University, Lanzhou, China; Zhejiang Agriculture and Forestry University: Zhejiang A and F University, CHINA

## Abstract

Active particulate organic carbon (POC) and inert mineral-associated organic carbon (MAOC) play critical roles in regulating and predicting global climate change. However, our understanding remains limited regarding how land-use changes differentially affect these two carbon fractions and their underlying driving mechanisms. In this study, we applied a physical fractionation approach to examine changes in the carbon content and mass fractions of particulate organic matter (POM) and mineral-associated organic matter (MAOM) following the conversion of grasslands to forested or abandoned lands on the Chinese Loess Plateau. This work provides the first systematic quantification of the trade-offs among different land-use types with respect to distinct soil carbon fractions in this region. The results revealed that land use significantly altered the distribution of carbon fractions. Although afforestation increased POC contents in both soil layers (9.70 and 7.82 g·kg ⁻ ¹, respectively), it markedly reduced the more stable MAOC contents (from 9.53 and 9.62 g·kg ⁻ ¹ to 7.53 and 6.08 g·kg ⁻ ¹ in the 0–0.15 m and 0.15–0.30 m layers, respectively) and MAOM mass fractions (from 77.46% to 68.16% and from 78.69% to 71.61%). In contrast, land abandonment led to an overall decline in both carbon fractions. Mechanistically, this study revealed distinct dominant drivers for POC and MAOC: soil physicochemical properties primarily governed POC variation (68.15%), whereas microbial communities controlled MAOC variation (67.01%). Notably, bacterial β-diversity exhibited a significant negative correlation with MAOC content, offering a new perspective on the role of microbes in carbon stabilization. In summary, grassland afforestation reduced soil carbon stability by inhibiting MAOC formation, while land abandonment decreased the total soil carbon pool. These findings suggest that maintaining natural grasslands may be the most effective approach for soil carbon conservation on the Loess Plateau, and that afforestation strategies should be carefully evaluated for their potential impacts on carbon stability.

## Introduction

Global soil organic carbon (SOC) stocks are crucial for regulating climate change and maintaining ecosystem sustainability. As the largest terrestrial carbon pool, SOC dynamics are increasingly recognized as critical for carbon-climate feedbacks and climate change mitigation strategies [1–4]. However, intensifying land-use change worldwide is fundamentally altering SOC dynamics, with significant implications for global carbon cycling and soil functioning. While some land-use changes, such as deforestation and urbanization, deplete SOC stocks [5–7], others, such as reforestation and grassland restoration, enhance carbon sequestration [8,9].

Understanding SOC responses to land-use change requires moving beyond total carbon measurements to resolve its distinct functional pools. SOC consists of components that differ in stability and turnover time, making it essential to distinguish among these pools to achieve accurate predictions of carbon cycling [10,11]. Physical fractionation methods commonly identify two key SOC components: particulate organic carbon (POC, > 53 μm) and mineral-associated organic carbon (MAOC, < 53 μm) [12,13]. These fractions operate under fundamentally different stabilization mechanisms and respond distinctly to environmental changes.

POC, composed mainly of partially decomposed plant residues, represents an active carbon pool with a relatively rapid turnover rates [8,14]. Its high accessibility to decomposers makes it a sensitive indicator of short-term SOC changes [15]. In contrast, MAOC forms through organo-mineral associations, resulting in a stable carbon pool characterized by longer residence times and greater resistance to microbial decomposition [16,17]. This stability renders MAOC crucial for long-term carbon sequestration and soil quality maintenance [18]. Therefore, quantifying these organic carbon fractions is therefore essential for advancing our understanding of terrestrial carbon cycling and assessing the sensitivity of SOC to variations in climate, land use, and management practices [10,19,20]. Furthermore, investigating how different land-use types influence POC and MAOC provides essential scientific insights into the mechanisms driving soil carbon storage and supports the development of effective climate change mitigation strategies [21].

Recent evidence indicates that POC and MAOC respond differently to land-use changes, yet the driving factors behind these divergent responses remain poorly understood, particularly in ecologically sensitive regions. Although vegetation, soil physicochemical properties, and microbial community diversity have been widely recognized as the three principal driving factors, most studies have examined their effects independently rather than in an integrated framework. In Argentina’s semiarid Chaco region, forest conversion to cropland resulted in greater POC losses compared to MAOC, with both fractions declining progressively over time [22]. The primary driving factors were changes in land use type, crop rotation composition, and crop biomass input. Al Shoumik et al. [23] conducted a multi-regional study across Europe and demonstrated that land-use practices t regulate the differential accumulation of POC and MAOC by regulating carbon-input pathways and substrate quality mediated by vegetation types. Their findings showed that forest soils tended to accumulate POC, whereas grasslands favored MAOC formation. Furthermore, Zhang et al. [24], through a meta-analysis of 266 global studies, revealed that land degradation generally reduced both fractions, while restoration increased them. Among the two, POC exhibited greater sensitivity, and variations in both carbon pools were largely governed by microbial biomass carbon. A more recent meta-analysis by Zhao et al. reported similar patterns. However, it remains unclear how vegetation, soil physicochemical properties, and microbial community diversity act as a co-varying continuum and jointly regulate the differential responses of POC and MAOC through their interactions. Despite these advances, how vegetation, soil properties, and microbial communities interact as a co-varying continuum to jointly regulate the contrasting responses of POC and MAOC remains unclear. This knowledge gap constrains our ability to predict and manage SOC dynamics under changing land-use patterns. In particular, this issue has not yet been systematically investigated in the ecologically fragile Loess Plateau region.

Chinas Loess Plateau, which has undergone extensive land use changes, including widespread conversion of natural grasslands to both afforested and abandoned lands, presents an ideal system for investigating these dynamics [25]. Although research in this region has yielded valuable insights, most studies remain limited to assessments of total soil organic carbon (SOC). For example, Zhu et al. [26] conducted a systematic evaluation of SOC stock dynamics under different land-use types in the Nangou watershed, highlighting the carbon-sequestration potential of natural vegetation. Similarly, Deng et al. [27] examined SOC accumulation rates during vegetation succession in the Ziwuling forest region and identified litter and fine-root inputs as the major driving factors. However, these studies did not explore SOC at the component level; they neither quantified the relative contributions of POC and MAOC nor elucidated the transformation pathways governed by carbon inputs. Consequently, the driving factors underlying soil-carbon sequestration under land-use change remain unclear, constraining the accurate assessment of soil carbon sinks and hindering the development of effective management strategies. Given the Plateau’s distinctive soil and climatic conditions, which may lead to carbon-stabilization patterns differing from those of other ecosystems, current assessments and management practices based solely on total SOC lack the necessary scientific precision. Based on these considerations, this study was designed to elucidate how the conversion of grassland to afforested or abandoned land affects SOC fraction dynamics and stability on the Loess Plateau. Specifically, we aimed to: (i) quantify the changes in POM and MAOM mass fractions, as well as the contents of POC and MAOC, across these land-use transitions; (ii) determine how these changes affect SOC stability by examining the relative contributions of POC and MAOC to total SOC; and (iii) identify the key biological and physicochemical factors controlling POC and MAOC responses to land-use change. We hypothesize that: (i) these land-use changes will alter the relative proportion of stable versus labile carbon pools, affecting overall SOC stability; and (ii) soil physicochemical properties and microbial communities will show different relative importance in controlling POC versus MAOC dynamics.

By addressing these objectives, our study provides new insights into how land use changes affect soil carbon stability through their differential impacts on carbon fractions. These findings have important implications for land management strategies aimed at enhancing soil carbon sequestration in ecologically sensitive regions worldwide.

## Materials and methods

### Study area

The study was located in the western Loess Plateau of China (35°53′N, 103°21′E), at elevations ranging from 1494 m to 3625 m. Between 1995 and 2020, the multi-year average temperature across the Loess Plateau ranged from −13°C to 16°C, and the annual precipitation ranged from 100 to 700 mm, showing an overall spatial pattern of increasing values from northwest to southeast [28]. The study area, Lanzhou City, experiences scarce rainfall, with an average annual precipitation of 324.85 mm, while the annual evaporation reaches as high as 1,486 mm. From 2000 to 2020, the regional temperature exhibited an overall increasing trend [29]. Other studies report that high-temperature days (daily maximum ≥ 35°C) increased sharply from only three (1980–1999) to 19 (2000–2009) and further to 23 (2010–2019) [30], indicating a significant increase in extreme heat frequency. The predominant soil type is calcareous, characterized by a loose, granular structure. Natural grasslands historically dominated this ecosystem, but significant land use changes occurred throughout the last century. Throughout the century, grasslands first underwent conversion to croplands—later abandoned due to rural-urban migration—and were then replaced by afforested lands as part of the national “Greening Project.” [31].

### Experimental design and field sampling

Field sampling was conducted in July 2020 along an east-facing slope of the Loess Plateau, with elevations ranging from 1830 m and 2100 m and slope gradients of 25°-40°. Sampling zones were established to representing three land-use types: natural grasslands, afforested lands (converted from grasslands), and abandoned lands (former croplands converted from grasslands). Sampling was further stratified across three elevation ranges: 1800–1900 m, 1900–2000 m, and 2000–2100 m.

A total of 53 plots were established: including 27 in grasslands, 21 in afforested lands, and 5 in abandoned lands. The vegetation in the grasslands was dominated by *Artemisia gmelinii Weber* ex Stechm., *Artemisia scoparia* Waldst. & Kitam., *Heteropappus altaicus* Novopokrov., *Artemisia capillaris* Thunb., *Peganum harmala* L., and *Medicago sativa* L. Afforested areas were primarily composed of Chinese arborvitae [*Platycladus orientalis* (L.) Franco], with understory species like *Melica przewalskyi* Roshev., *Poa versicolor* Besser, *Stipa grandis* P.A. Smirn., and *Peganum harmala* L. Abandoned lands (approximately ten years since abandonment) were characterized by *Festuca ovina* L., *Stipa capillata* L., and *Achnatherum splendens* (Trin.) Nevski [25].

Within each plot, we established five 0.50 m × 0.50 m quadrats (four corners and center) for vegetation and soil sampling. We collected:

- Aboveground herbaceous biomass (AGB) using harvest methods
- Belowground herbaceous biomass (BGB) using a 0.01 m diameter auger
- Soil samples at two depths (0–0.15 m and 0.15–0.30 m)

In afforested plots, we additionally recorded tree metrics including number of trees, height, and diameter at breast height (Table 1).

**Table 1:** Basic characteristics of the afforested land.

Altitude (m)	Average diameter at breast height (m)	Average tree height (m)	Number of trees (ha^-1^)
1800 ~ 1900	0.22	3.90	1005 ~ 1410
1900 ~ 2000	0.26	5.09	
2000 ~ 2100	0.27	5.77	

### Soil physicochemical analysis

Soil physicochemical analyses were performed on all collected samples from both soil depths. Soil water content was determined gravimetrically, pH was measured in a 1:2.5 soil-to-water suspension, and SOC was analyzed using the potassium dichromate oxidation method [32]. Total soil nitrogen was quantified by the Kjeldahl method [33], while ammonium nitrogen (${\text{NH}}_{\text{4}}^{+}$-N) and nitrate nitrogen (${\text{NO}}_{\text{3}}^{-}$-N) were measured using continuous-flow analysis following KCl extraction. Total and available phosphorus were analyzed following Olsen and Sommers [34]. Particle size distribution was determined using the pipette method after H_2_O_2_ treatment and sodium hexametaphosphate dispersion, categorizing particles into clay (<2 μm), silt (2–50 μm), fine sand (50–250 μm), and coarse sand (250–2000 μm) fractions. The vegetation biomass and basic physico-chemical properties of soil under different land-use types are presented in Table 2.

**Table 2:** Vegetation biomass and soil physicochemical properties across different land-use types (most of the data from Zhang et al. [25].

	Soil depth (m)	Grassland	Afforested land	Abandoned land
AGB (g m^‑2^)		306.10 ± 123.18 b	12617.67 ± 3848.90 a	242.59 ± 124.87 b
BGB (g m^‑2^)	0-0.15	168.65 ± 104.19 b	1927.29 ± 532.38 a	155.18 ± 133.67 b
	0.15-0.30	111.13 ± 116.30 b	1826.10 ± 493.63 a	39.16 ± 27.76 b
SWC (%)	0-0.15	11.43 ± 1.70 a	12.60 ± 5.44 a	13.18 ± 4.64 a
	0.15-0.30	11.35 ± 1.89 a	9.39 ± 3.78 b	12.41 ± 2.53 a
pH	0-0.15	8.18 ± 0.16 b	7.94 ± 0.29 c	8.30 ± 0.21 a
	0.15-0.30	8.20 ± 0.14 b	7.93 ± 0.28 c	8.33 ± 0.18 a
SOC (g·kg^‑1^)	0-0.15	13.75 ± 3.55 a	14.25 ± 7.22 a	6.64 ± 1.35 b
	0.15-0.30	11.91 ± 3.071 a	9.75 ± 4.42 b	4.19 ± 1.00 c
STN (g·kg^‑1^)	0-0.15	1.30 ± 0.77 a	0.66 ± 0.53 b	0.43 ± 0.23 b
	0.15-0.30	1.29 ± 0.79 a	0.58 ± 0.45 b	0.37 ± 0.34 b
STP (g·kg^‑1^)	0-0.15	0.35 ± 0.19 a	0.28 ± 0.17 b	0.38 ± 0.17 a
	0.15-0.30	0.30 ± 0.175 a	0.28 ± 0.16 a	0.30 ± 0.14 a
${\text{NH}}_{\text{4}}^{+}$-N (mg·kg^‑1^)	0-0.15	6.98 ± 1.71 a	5.70 ± 2.98 a	2.52 ± 1.23 b
	0.15-0.30	6.26 ± 1.79 a	4.52 ± 2.87 b	1.36 ± 1.77 c
${\text{NO}}_{\text{3}}^{-}$-N (mg·kg^‑1^)	0-0.15	9.73 ± 7.12 b	18.72 ± 21.42 a	5.21 ± 3.42 b
	0.15-0.30	6.56 ± 4.84 b	8.68 ± 5.48 a	1.61 ± 0.57 c
AP (mg·kg^‑1^)	0-0.15	4.28 ± 1.12 b	4.77 ± 1.79 a	4.23 ± 0.97 b
	0.15-0.30	3.75 ± 1.04 b	4.31 ± 1.71 a	3.39 ± 0.94 b
Clay (%)	0-0.15	6.49 ± 0.51 a	5.98 ± 0.62 b	4.89 ± 0.41 c
	0.15-0.30	6.33 ± 0.61 a	5.67 ± 0.64 b	4.89 ± 0.34 c
Silt (%)	0-0.15	46.45 ± 2.39 a	40.30 ± 4.32 b	36.86 ± 3.10 c
	0.15-0.30	47.61 ± 2.69 a	39.46 ± 4.21 b	37.29 ± 2.72 c
Fine (%)	0-0.15	46.13 ± 2.67 c	51.65 ± 4.45 b	57.54 ± 3.20 a
	0.15-0.30	44.69 ± 2.35 c	52.91 ± 4.53 b	57.63 ± 3.08 a
Coarse (%)	0-0.15	0.92 ± 0.50 b	2.00 ± 1.43 a	0.70 ± 0.81 b
	0.15-0.30	1.36 ± 2.15 b	1.94 ± 1.142 a	0.17 ± 0.33 c

The use of different lowercase letters denotes statistically significant variations (*p* ≤ 0.05) among the three land use types. AGB: aboveground herbaceous biomass; BGB: belowground herbaceous biomass; SWC: soil water content; SOC: soil organic carbon; STN: soil total nitrogen; STP: soil total phosphorus; ${\text{NH}}_{\text{4}}^{+}$-N: Soil ammonium nitrogen; ${\text{NO}}_{\text{3}}^{-}$-N: soil nitrate nitrogen; AP: soil available phosphorus.

### Physical fractionation and carbon analysis

Soil organic matter fractionation was performed following the procedures described Cambardella and Elliott [35] and Cotrufo et al. [36]. For each land-use type at each elevation segment, nine soil samples were randomly selected for analysis. The protocol involved dispersing 10 g of air-dried soil (< 2 mm) in a 0.5% sodium hexametaphosphate solution, shaking for 18 h at 90 rpm, and passing through a 53 μm sieve, followed by rinsing with deionized water. The material retained on the sieve (POM) and the filtrate (MAOM) were dried at 60°C, and their carbon contents were determined using potassium dichromate oxidation method to obtain POC and MAOC contents.

### Microbial community analysis

Surface soil samples (0–0.15 m) were analyzed for microbial community composition following the method described by Zhang et al. [25]. Total genomic DNA was extracted using the Fast DNA SPIN Kit for Soil, and the bacterial 16S rRNA gene V3-V4 regions were sequenced on an Illumina MiSeq platform. Bacterial α-diversity was assessed using the Shannon and Simpson indices, while β-diversity was evaluated based on Bray-Curtis dissimilarity matrices. Community functional potential was predicted using PICRUSt, focusing on genes associated with carbon cycling. These microbial parameters were integrated with soil physicochemical data to explore their relationships with carbon fraction dynamics.

### Statistical analysis

A comprehensive statistical framework was applied to analyze carbon-fraction dynamics across different land-use types. One-way ANOVA followed by Least Significant Difference (LSD) tests was used to evaluate differences in carbon fractions among land-use types, while Mann-Whitney U tests evaluated differences between soil depths within sites. To account for spatial heterogeneity, linear mixed models were implemented with land-use type as a fixed effect and sampling sites as random effects. Key factors influencing POC and MAOC were identified using redundancy analysis (RDA) after testing for multicollinearity among vegetation, soil, and microbial variables. Mechanistic pathways were further explored throught variance partitioning and structural equation modeling (SEM) to elucidate the direct and indirect effects of environmental factors on carbon fractions. All statistical analyses were conducted using SPSS 22.0 and R 4.2.3 (vegan package), with statistical significance set at *p* ≤ 0.05. Data visualization was performed in Origin 9.0 refined using the using ggplot2 and ggrepel packages in R 4.2.3to produce detailed graphical representations of the results.

## Results

### Distribution of POM and MAOM under different land use types

**Fig 1:**
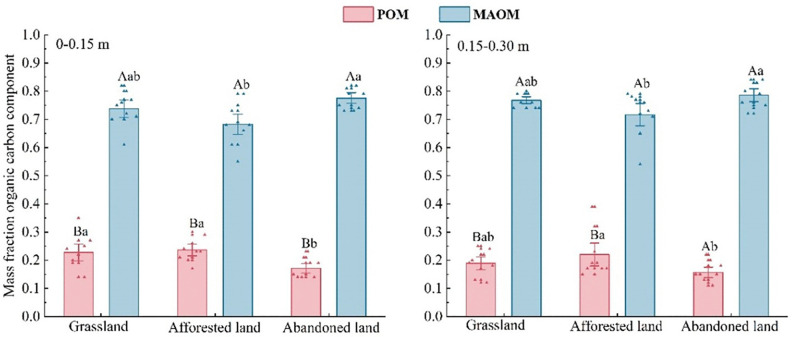
Proportions of POM and MAOM under different land use types. Significant differences (*p* ≤ 0.05) are indicated by different letters. Lowercase letters are assigned to differences among land use types for POM and MAOM quality fractions, and uppercase letters to differences between POM and MAOM within a land use type.

Land-use type significantly influenced the mass fractions of particulate organic matter (POM) and mineral-associated organic matter (MAOM) in soil samples. In the surface layer (0–0.15 m), POM fractions were significantly higher in grasslands (22.73%) and afforested lands (23.50%) than in abandoned lands (17.10%). This pattern persisted in the subsurface layer (0.15–0.30 m), where afforested lands maintained the highest POM fraction (22.19%), significantly exceeding that of abandoned lands (15.58%), but showing no significant difference from grasslands (18.96%). MAOM dominated the total soil organic matter across all land use types and depths, with proportions significantly higher than those of POM. Notably, abandoned lands showed the highest MAOM proportions (77.46% and 78.69% in the surface and subsurface layers, respectively), significantly exceeding those in afforested lands (68.16% and 71.61%, respectively) (Fig 1). No significant depth-related differences in POM or MAOM proportions were observed among land-use types, as detailed in S2 Table in S1 File.

### POC and MAOC content variations across land use types

**Fig 2:**
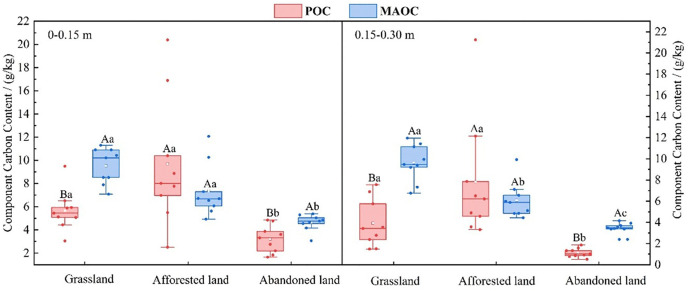
POC and MAOC contents under different land use types. Significant differences (*p* ≤ 0.05) are indicated by different letters. Lowercase letters are used to denote differences among land-use types for POC and MAOC quality fractions, and uppercase letters are used to denote differences between POC and MAOC within a land-use type.

The contents of both POC and MAOC exhibited distinct patterns across land-use types and soil depths. POC content was significantly higher in afforested lands (9.70 g/kg and 7.82 g/kg in surface and subsurface layers, respectively) and grasslands (5.66 g/kg and 3.92 g/kg) than in abandoned lands (3.21 g/kg and 1.13 g/kg). Similarly, MAOC content was greater in both afforested lands (7.53 g/kg and 6.08 g/kg) and grasslands (9.53 g/kg and 9.62 g/kg) compared with abandoned lands (4.56 g/kg and 3.48 g/kg). A notable vertical stratification was observed in abandoned lands, where both POC and MAOC contents were significantly higher in the surface layer compared to the subsurface layer. In grasslands and abandoned lands, MAOC content exceeded POC content at both depths, while afforested lands showed no significant difference between these fractions (Fig 2).

**Fig 3:**
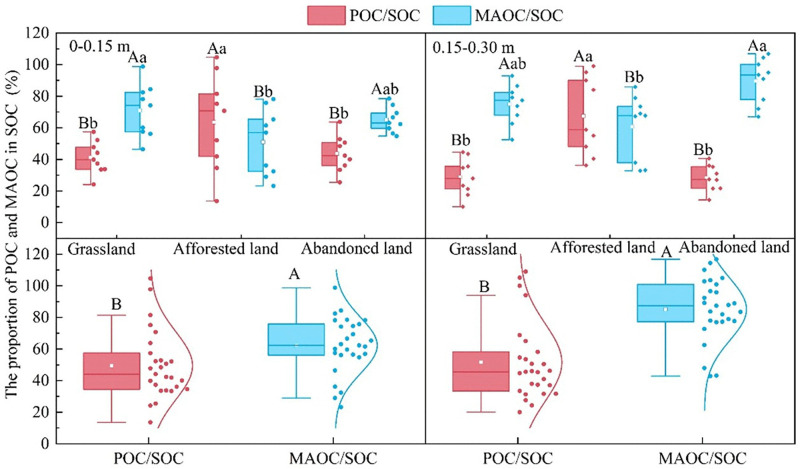
POC/SOC and MAOC/SOC under different land use types. Significant differences (*p* ≤ 0.05) are indicated by different letters. Lowercase letters are used to denote differences among various land-use types, and uppercase letters are used to denote differences between POC/SOC and MAOC/SOC within the same land-use type Fig 4.

The relative contributions of POC and MAOC to SOC varied significantly among land-use types, revealing distinct patterns of carbon stabilization. The MAOC/SOC ratio generally exceeded the POC/SOC ratio across the study area, indicating the dominance of MAOC in the soil carbon pool. This pattern was particularly pronounced in natural grasslands and abandoned lands, where MAOC contributed substantially more to SOC than POC. However, afforested lands showed a contrasting pattern, with POC/SOC ratios exceeding MAOC/SOC ratios, suggesting a shift in carbon storage dynamics following afforestation. These distinct patterns highlight the differential impacts of land-use change on carbon pool composition ability of soil carbon pools (Fig 3).

**Fig 4:**
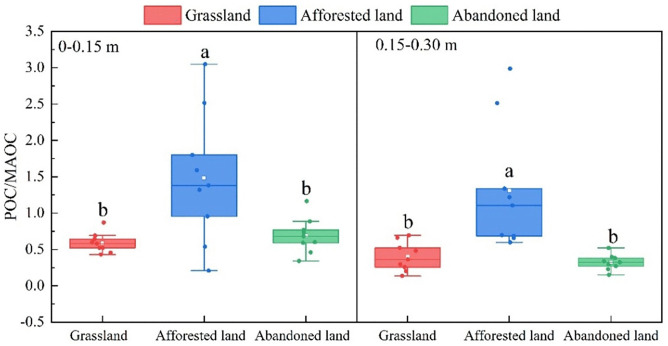
Characteristics of POC/MAOC under different land uses. Different lowercase letters denote statistically significant differences among land use types. Different letters indicate significant differences at *p* ≤ 0.05.

 To further assess carbon stability among different land use types, we analyzed POC/MAOC ratios, which serve as indicators of organic carbon dynamics and stabilization. Afforested lands exhibited significantly higher POC/MAOC ratios compared to both grasslands and abandoned lands, indicating potentially lower carbon stability in these converted ecosystems (Fig 4). 

### Factors controlling POC and MAOC dynamics

The linear mixed model analysis revealed that variations in POC and MAOC contents were primarily determined by land-use type (fixed effects) rather than sampling location (random effects) at both soil depths. This analysis showed high marginal (R²m) and conditional (R²c) R-squared values, particularly for MAOC (R²m = 0.76, R²c = 0.82 at 0.15–0.30 m depth), indicating strong land-use effects on the distribution of carbon fraction (Table 3).

**Table 3:** Fixed effects (land-use type) and random effects (sample site) on POC and MAOC contents.

Parameters	Soil depth (m)	Fixed effects			Random effects	R²m marginal	R² conditional
		F	df	*p*	*P*		
POC	0-0.15	8.08	19	**	ns	0.35	0.47
	0.15-0.30	10.81	19	***	ns	0.37	0.60
MAOC	0-0.15	23.98	19	***	ns	0.60	0.72
	0.15-0.30	49.17	19	***	ns	0.76	0.82

**Fig 5:**
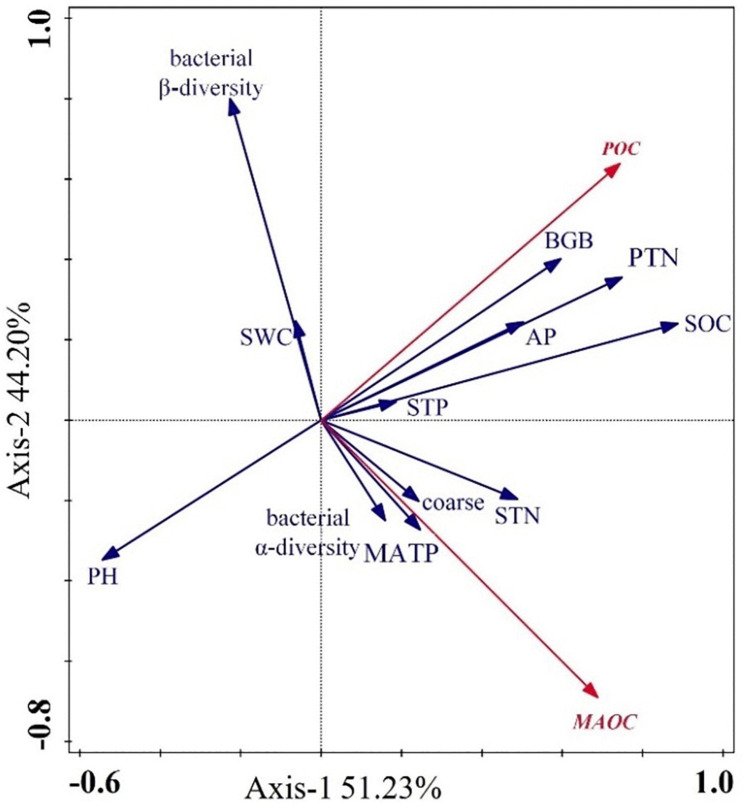
Redundancy analysis ranking of POC and MAOC with environmental factors. PTN: particulate nitrogen, MAON: mineral-associated nitrogen, MATP: mineral-associated phosphorus. Explanatory variables are shown in blue and response variables are shown in red. The length of the arrow and the origin reflects the degree of correlation.

Statistical significance was denoted as follows: * for *p* ≤ 0.05, ** for *p* ≤ 0.01, *** for *p* ≤ 0.001, and ‘ns’ for not significant (*p* > 0.05). Redundancy analysis (RDA) identified the key factors influencing the distribution of POC and MAOC, with the first two axes explaining 95.43% of the total variance (51.23% and 44.20%, respectively) (Fig 5). Among the environmental variables analyzed, belowground biomass (BGB), particulate nitrogen (PTN), soil organic carbon (SOC), bacterial β-diversity, and pH emerged as the primary factors controlling POC and MAOC dynamics (Table 4). The RDA biplot revealed distinct associations between these environmental factors and carbon fractions, indicating clear separation in their influence patterns.

### Mechanistic pathways of POC and MAOC formation

**Fig 6:**
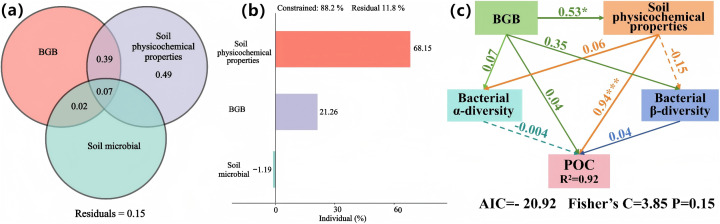
Contribution of biological and environmental factors to POC content variation and their impact mechanisms. Statistical significance was denoted as follows: * for *p* ≤ 0.05, ** for *p* ≤ 0.01, *** for *p* ≤ 0.001.

Our analysis revealed distinct mechanistic pathways controlling POC and MAOC formation. Environmental and biological indicators collectively explained 88.2% of the total variation in POC content variation (Fig 6b), with soil physicochemical properties contributing the largest proportion (68.15%). Belowground biomass individually accounted for 21.26% of POC variation (Fig 6b), and when combined with other soil physicochemical factors, explained 39% of POC variation through indirect pathways (Fig 6a, c).

**Fig 7:**
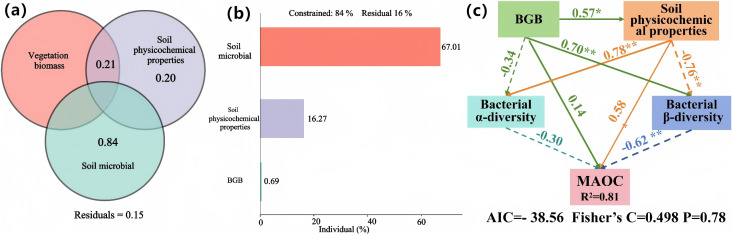
Contribution of biological and environmental factors to MAOC and their impact mechanisms. Statistical significance was denoted as follows: * for *p* ≤ 0.05, ** for *p* ≤ 0.01, *** for *p* ≤ 0.001.

For MAOC content, biological and environmental factors together explained 83.97% of the total variance, with soil microorganisms being contributing the largest proportion (67.01%) (Fig 7b). The analysis further revealed that belowground biomass indirectly influenced MAOC content through its effects on soil physicochemical properties and bacterial β-diversity (Fig 7c). Additionally, soil physicochemical properties showed both direct and indirect effects on MAOC through their influence on bacterial α-diversity and β-diversity (Fig 7c).

## Discussion

### Land use change fundamentally alters soil carbon fraction distribution

Our findings reveal distinct responses of soil carbon fractions to land-use change, supporting our Hypothesis 1. These results have important implications for soil carbon stability and sequestration potential. The physical fractionation procedure achieved over recovery rates exceeding 94% across all land-use types and depths (S1 Table in S1 File), confirming the reliability of our approach in distinguishing soil organic matter pools. The dominance of MAOM across all land-use types is consistent with previous findings [36,37], indicating the prevalence of stable carbon forms in these soils, yet this balance is significantly altered by land-use transitions.

The conversion of grasslands to afforested lands markedly reduced MAOM proportions while increasing POM (Fig 1), suggesting a fundamental shift in carbon stabilization mechanisms. This shift can be attributed to several interacting factors. First, the lower soil pH observed in afforested lands (S1 Table in S1 File) likely disrupts microbial community functioning, constraining the microbial transformation processes critical for MAOC formation [38,39]. Second, soil acidification reduces mineral surface availability for organic matter adsorption [40], thereby limiting MAOM formation. Additionally, the chemical composition of cypress litter, rich in recalcitrant compounds such as lignin, cutin, and suberin [41], may further inhibit MAOM formation while contributing to POM accumulation.

The increased POM in afforested lands, driven by high above- and below-ground biomass inputs (S1 Table in S1 File), and minimal soil disturbance, likely enhances macroaggregate formation that facilitates POM retention and protection [42]. However, this shift toward POM dominance raises concerns about carbon stability, as POM is more labile and thus more susceptible to microbial decomposition and environmental perturbations [14,43].

### Mechanisms controlling carbon fraction dynamics

Our analysis reveals complex regulatory mechanisms for POC and MAOC formation, supporting Hypothesis 2. Contrary to prevailing views that emphasize vegetation as the dominant driver of POC dynamics [44], we found that soil physicochemical properties accounted for 68.15% of the variation in POC content. Belowground biomass influenced POC primarily through indirect pathways by modifying soil physicochemical properties (Fig 6), consistent with recent findings highlighting the critical role of root-soil interactions in carbon cycling [45,46].

The predominant of microbial control over MAOC formation (67.01% contribution) aligns with the emerging understanding of microbial-mineral interactions as key processes governing carbon stabilization [47,48]. However, our finding of a negative correlation between bacterial β-diversity and MAOC content reveals a previously unrecognized complexity (Fig 5). This relationship likely reflects the high energetic costs associated with MAOC decomposition and formation [49,50], where diverse bacterial communities may preferentially utilize more accessible carbon sources due to competitive pressures. This interpretation is further supported by recent evidence showing that MAOC resistance to microbial decomposition increases with the strongest physical protection mechanisms [51,52].

### Soil chemical properties as master variables in carbon stabilization

Soil pH emerged as a critical factor influencing both POC and MAOC dynamics (Fig 5, Table 4), consistent with its role as a master variable in soil processes. Under acidic conditions, reduced microbial decomposition and inhibited lignin degradation lead to increased POC retention [53]. This accumulation is further enhanced by declines in microbial biomass carbon and reductions in the activity of carbon hydrolytic enzyme in acidic soils [54,55]. The negative relationship between pH and MAOC can be attributed to pH-dependent changes in mineral surface charge and organic ligand interactions [56,57]. As pH increases, carbon stabilization mechanisms shift from organic-metal complexations to associations with short-range-order minerals and calcium complexes [58], thereby altering MAOC formation dynamics.

**Table 4:** Explanation of total variation in POC and MAOC contents by environmental factors.

	RDA1	RDA2	r^2^	Pr (> r)
BGB	0.82	0.57	0.50	0.001**
PTN	0.90	0.44	0.66	0.00***
MATP	0.67	−0.75	0.13	0.40
SOC	0.96	0.27	0.80	0.00***
STN	0.93	−0.37	0.26	0.09
STP	0.97	0.25	0.04	0.76
AP	0.90	0.45	0.30	0.07
SWC	−0.24	0.97	0.06	0.59
pH	−0.84	−0.55	0.40	0.02*
Coarse	0.77	−0.64	0.09	0.44
Bacterial α-diversity	0.53	−0.85	0.08	0.51
Bacterial β-diversity	−0.264	0.96	0.66	0.00***

Statistical significance was denoted as follows: * for *p* ≤ 0.05, ** for *p* ≤ 0.01, *** for *p* ≤ 0.001.

### Implications for carbon sequestration and land management

The differential responses of carbon fractions to land-use change have significant implications for ecosystem management and carbon sequestration strategies. Our findings challenge the widespread assumption that afforestation universally enhances soil carbon stability. While afforestation increases total carbon inputs, the shift toward POC dominance compromises long-term carbon stability, in line with recent studies questioning the sustainability of afforestation-based carbon sequestration [59,60]. Afforestation-induced soil acidification is likely a key bottleneck limiting the formation of MAOC. Accordingly, afforestation in the study region should proceed with caution. Future programs must move beyond simply “planting trees” to incorporate continuous monitoring and precise regulation of soil pH (e.g., selecting non-acidifying tree species and judiciously applying soil amendments) as core management measures, thereby ensuring that additional carbon inputs are effectively converted into long-term, stable carbon stocks. Moreover, in acidic or ecologically fragile areas, near-natural restoration options—such as maintaining or establishing natural grasslands—should be prioritized.

Abandoned lands present a distinct challenge, where the combined effects of previous cultivation history and current vegetation dynamics create a complex carbon cycling scenario. The significantly lower POC and MAOC contents in abandoned lands, compared to other land-uses types (Fig 2), are consistent with findings from similar ecosystems [61,62]. This reduction results from the disruption of soil aggregates during previous cultivation, which diminished the physical protection of organic carbon and accelerated the decomposition of the active carbon pool [63,64]. Moreover, the sparse vegetation cover in abandoned lands, as indicated by low biomass measurements (S1 Table in S1 File), not only limits the input of new organic matter input but also increases susceptibility to erosional losses [65,66]. Therefore, abandoned lands should not be treated passively as natural recovery areas; instead, they should be designated as “priority intervention zones” for carbon sequestration. Management should focus on two key strategies: restoring soil structure by introducing deep-rooted species, and accelerating vegetation establishment to capture new carbon inputs and mitigate erosion

### Mechanistic insights and future research directions

Our findings provide novel insights into the microbial regulation of soil carbon fractions. The dominance of soil microorganisms in controlling MAOC variation (67.01%) supports emerging theories regarding microbial residues contributions to soil carbon storage [67,68]. However, the complex relationship between bacterial diversity and carbon fraction dynamics suggests that microbial community composition may be as important as diversity in determining carbon stabilization outcomes [69,70].

The role of belowground biomass in indirectly influencing MAOC through bacterial β-diversity and soil properties (Fig 7) underscores the need for integrated understanding of plant-soil-microbe interactions. Recent studies have highlighted the crucial role of root exudates and microbial products in forming stable soil organic matter [71,72], but the pathways through which these interactions affect different carbon fractions require further investigation.

### Global context and future perspectives

Our findings have broader implications for global carbon cycling models and land management policies, particularly in regions experiencing similar land-use transitions. The distinct responses of POC and MAOC to land-use change suggest that carbon sequestration potential cannot be accurately predicted without accounting for fraction-specific dynamics [15]. The observed trade-offs between carbon quantity and stability across different land uses highlight the need for management strategies that optimize both aspects.

Future research should address several key knowledge gaps. First, the long-term stability of carbon fractions under projected climate change scenarios requires investigation, particularly in regions experiencing increasing aridity. Second, a deeper understanding of how microbial community composition and function influence carbon fraction formation is needed. Finally, developing management strategies that enhance MAOC formation while maintaining ecosystem productivity remains a critical challenge.

## Conclusions

Our systematic study of soil organic carbon (SOC) fractions in the Loess Plateau reveals important patterns in carbon dynamics under land-use changes. The conversion of grasslands to afforested lands resulted in a decrease in the proportion of stable MAOM and an increase in the proportion of labile POM, suggesting that the carbon sequestration potential of afforestation strategies in similar ecological contexts may be overestimated and requires careful evaluation. In contrast, the conversion of grasslands to abandoned lands caused significant declines in both POC and MAOC contents, indicating severe degradation of soil carbon storage capacity.

Mechanistically, soil physicochemical properties primarily governed POC variations, whereas microbial communities significantly influenced MAOC formation. Land-use change operates mainly by altering vegetation and soil physicochemical conditions, thereby reshaping microbial diversity and ultimately driving differences in POC and MAOC. Therefore, effective soil carbon management must account for both abiotic and biotic factors.

Based on the results of this study, we recommend that soil carbon management on the Loess Plateau focus on the protection of natural grasslands and the optimization of afforestation practices—maintaining appropriate pH levels, minimizing human disturbance, and retaining litter. For abandoned lands with high ecological risks, systematic measures for soil and water conservation and carbon pool restoration should be prioritized. In implementing this strategy, it is crucial to fully recognize the role of soil microorganisms and incorporate their community structure and dynamic changes into management and decision-making frameworks.

This study advances our understanding of how land-use changes regulate soil carbon stability through their differential impacts on carbon fractions. Future research should focus on long-term monitoring of carbon fraction dynamics under changing climatic conditions and developing precise management strategies that simultaneously enhance carbon stocks and maintain carbon stability.

## Supporting information

S1 File S1 Table. Soil Mass recovery (%) under different land use types. **S2 Table.** The differences in POC, MAOC and the proportion of POM and MAOM at soil depth under different land use types. **S3 Table.** Key factors influencing POC and MAOC content. This dataset presents the key factors—including vegetation characteristics, soil physicochemical properties, and microbial diversity—identified as influencing POC and MAOC content following collinearity diagnosis.
(ZIP)
