# Supplementary material for: Natural grasslands converted to afforested lands and abandoned lands decreasing soil carbon stability and storage, respectively, in the China’s Loess Plateau
Source: PLoS One. 2025 Nov 18;20(11):e0335924. doi: 10.1371/journal.pone.0335924 (PMC12626324; doi:10.1371/journal.pone.0335924)
Supplement: S1 File — Soil Mass recovery (%) under different land use types. S2 Table. The differences in POC, MAOC and the proportion of POM and MAOM at soil depth under different land use types. S3 Table. Key factors influencing POC and MAOC content. This dataset presents the key factors—including vegetation characteristics, soil physicochemical properties, and microbial diversity—identified as influencing POC and MAOC content following collinearity diagnosis. (ZIP) [file pone.0335924.s001.zip › Supporting Information/S1 Table.docx]

S1 Table. Soil Mass recovery (%) under different land use types.

| Soil depth （m） | Grassland | Abandoned land | Afforested land |
| --- | --- | --- | --- |
| 0-0.15 | 96.33±1.73 | 94.56±1.01 | 94.50±2.50 |
| 0.15-0.30 | 95.56±2.56 | 94.44±1.74 | 94.88±1.96 |
